# Ampicillin Vs. Penicillin for In Utero Therapy

**DOI:** 10.1155/S1064744996000105

**Published:** 1996

**Authors:** Douglas D. Glover, David Lalka, Gilles R. G. Monif

**Affiliations:** 1 Department of Obstetrics and Gynecology West Virginia University School of Medicine Robert C. Byrd Health Sciences Center Morgantown WV USA; 2 Department of Basic Pharmaceutical Sciences West Virginia University School of Pharmacy Robert C. Byrd Health Sciences Center Morgantown WV USA; 3 Department of Obstetrics and Gynecology Creighton University School of Medicine 601 No. 30th Street Suite 4700 Omaha NE 68131 USA

## Abstract

The pharmacokinetics of penicillin G and ampicillin are reviewed as they pertain to their potential use in in vitro therapy.


Infectious Diseases in Obstetrics and Gynecology 4:43–46 (1996)
© 1996 Wiley-Liss, Inc.

### Ampicillin Vs. Penicillin for In Utero Therapy

**Douglas D. Glover, David Lalka, and Gilles R.G. Monif**

*Department of Obstetrics and Gynecology, West Virginia University School of Medicine (D.D.G.), and Department of Basic Pharmaceutical Sciences, West Virginia University School of Pharmacy (D.D.G., D.L.), Robert C. Byrd Health Sciences Center, Morgantown, WV; Department of Obstetrics and Gynecology, Creighton University School of Medicine, Omaha, NE (G.R.G.M.)*

ABSTRACT

The pharmacokinetics of penicillin G and ampicillin are reviewed as they pertain to their potential use in in vitro therapy. © 1996 Wiley-Liss, Inc.

KEY WORDS

Pharmacokinetics, benzylpenicillin, penicillin G, β-lactams, GBS prophylaxis

The molecular structure of the penicillins consists of a thiazolidine ring connected to a β-lactam ring attached to a side chain. The side chain determines many of the pharmacologic characteristics of a given penicillin. The presence of an amino group on the benzyl side chain distinguishes ampicillin from benzylpenicillin (penicillin G), which is one example of the many chemical variants of penicillin resulting from alterations of this side chain attached to the basic 6 amino-penicillinoic acid molecule.

Recently, the issue of the relative superiority of penicillin G vs. ampicillin for in utero therapy has been raised. Amstey and Gibbs[1] wrote an editorial opinion stating that the "favorable pharmacokinetics of penicillin G and its narrower and specific spectrum make this a better choice for group B streptococcal (GBS) prophylaxis than ampicillin. . . ." This editorial opinion has stimulated a growing ambiguity about the peripartal use of ampicillin. The purpose of this review is to examine the key points of these contentions and, in so doing, clarify the issue of ampicillin vs. penicillin G for in utero therapy and GBS prophylaxis.

### LITERATURE REVIEW

A review of the literature on penicillin G and ampicillin reveals a dearth of pharmacokinetic data on penicillin G in pregnant subjects and extensive data on ampicillin in such patients.[2–9] A partial explanation emanates from the fact that penicillin G was widely used prior to the evolution of pharmacokinetics into a distinct discipline. When ampicillin was introduced in the early 1960s, pharmacokinetics was the "new kid on the block," thus having the benefit of investigation through these newly evolved scientific methods. Being the focus of attention in the literature, ampicillin came to be considered the β-lactam of choice and the utilization of penicillin G gradually faded.

### THEORETICAL FACTORS GOVERNING TRANSPORT OF ANTIBIOTICS ACROSS THE PLACENTAL BARRIER

Anatomically, the placenta is composed of ectodermal cells, a basement membrane, mesodermally derived tissue, another basement membrane, and fetal endothelial cells.[3] The only other embryologic structure in the human body derived from ectoderm

Address correspondence/reprint requests to Dr. Gilles R.G. Monif, Department of Obstetrics and Gynecology, Creighton University School of Medicine, 601 No. 30th Street, Suite 4700, Omaha, NE 68131.

Received December 18, 1995
Accepted December 19, 1995

Review Article and mesoderm as opposed to mesoderm and endoderm is the choroid plexus. Not surprisingly, the antibiotic transports across these 2 membranes strongly parallel each other. The factors governing antibiotic transport across the placental are 1) molecular size, 2) degree of protein binding, 3) degree of ionization, and 4) lipid solubility.[5]

### Molecular Size

The comparability of the size of penicillin G and ampicillin is such that this factor per se is not a significant determinant for differential transport of these 2 antibiotics.

### Degree of Protein Binding

Penicillin G is moderately bound to albumin in the sera of normal and pregnant subjects (65% bound compared with ampicillin's 20%).[3,5,7] To be pharmacologically active, an antibiotic must exist in its unbound state. The amount of antibiotic available for transport in a given time is a direct function of the concentration of free drug. Once the antibiotic is removed by transport, the equilibrium between bound and unbound drug is reestablished. The free drug entering the fetal vascular compartment and amniotic fluid is once again subjected to similar protein binding interactions which reduce the amount of antibiotic available for biologic activity. One of the keys to effective in utero therapy concerns how much pharmacologically active antibiotic appropriate to the bacteria in question can be delivered in a relatively limited time.

### Degree of Ionization

Both penicillin G (pKa = 2.8 for its free carboxylic-acid group) and ampicillin (pKa = 2.5 for its free carboxylic-acid group and pKa = 7.2 for its free amino group) are highly ionized (>99.9%) under physiologic conditions.[10] Thus, this factor has little impact on defining the relative rate of placental transfer.

### Lipid Solubility

Both penicillin and ampicillin antibiotics exhibit only moderate lipophilicity. The oil/water partition coefficients ($K_{o/w}$) are 0.55 and 0.16 for penicillin G and ampicillin, respectively (isobutanol vs. pH = 7.4 aqueous buffer).[11] However, in general, a difference of a factor of 3–4 in $K_{o/w}$ will have only moderate effects on drug permeability. Fundamentally, these characteristics simply slow the rate of placental transfer but do not prevent such transfer.

### COMPARATIVE PHARMACOKINETICS OF AMPICILLIN AND PENICILLIN G IN PREGNANCY

### Intravenous Administration

A review of the literature failed to identify any comparative pharmacokinetic studies of ampicillin and penicillin G in pregnant subjects receiving both drugs intravenously. Hence, direct intraindividual comparison of parameters such as volume of distribution, clearance, and half-life has not been made. Indeed, a recent review of this topic suggested that much of our knowledge of penicillin G pharmacokinetics in pregnancy is based on a number of very early and semiquantitative reports.[2]

### Intramuscular Administration

In what is perhaps the most comprehensive comparative study of these 2 drugs, identical intramuscular doses were given at various times during pregnancy with maternal, fetal, and amniotic-fluid sampling.[4] Ampicillin appears to yield consistently higher (approximately 50%) maternal and fetal serum concentrations than penicillin G. However, its level in amniotic fluid is substantially lower than that of penicillin G during the first 2 h following administration. After 3 h, superior and ultimately therapeutic concentrations of ampicillin appear in the amniotic fluid.

### Ampicillin Pharmacokinetics

In contrast to penicillin G, the pharmacokinetics of ampicillin have been studied thoroughly.[2,8,9] It has been established that pregnant women exhibit renal clearances and total body clearances that are about 50% greater than those under control conditions.[6] These control studies were performed 3–12 months after delivery when normal menstruation had resumed and breast-feeding had ceased. The oral bioavailability was very similar during pregnancy and 3–12 months postpartum (approximately 50%), and the recovery of the drug in the urine was essentially unaffected by pregnancy.[6,7] Furthermore, studies of the reproducibility of ampicillin levels following repeated administration of the drug to the same subject have demonstrated the dose proportionality of the area under the plasma-concentration time curve. Studies of the serum concentration in the mother following the administration of the drug into the amniotic fluid have been performed.[9]

Finally, MacAulay et al.[9] studied the transference of ampicillin within the maternal serum, fetal serum, and amniotic-fluid compartments. A transfer occurs from mother to fetus within minutes, but the antibiotic concentrations in the amniotic fluid are minimal for 60 min and potentially therapeutic levels for susceptible bacteria are not achieved until 90 min has elapsed. Therefore, the amniotic fluid appears to become a depository for the ampicillin.

### USE OF PENICILLIN G VS. AMPICILLIN FOR GBS PROPHYLAXIS

### Spectrum of Susceptibility

Once pharmacologically significant levels of an antibiotic are attained in the fetal compartment, the key question governing the biologic effectiveness is the spectrum of susceptibility of the organism involved. Both penicillin G and ampicillin share a common spectrum of activity against gram-positive bacteria. However, minor differences exist. For example, the minimum inhibitor concentrations (MIC) for ampicillin are lower for enterococci and *Listeria monocytologenes*, whereas penicillin G is more effective than ampicillin for hemolytic streptococci.[6] This superior MIC for the β-hemolytic streptococci does not necessarily translate into biologic significance. The clear difference between penicillin G and ampicillin is the latter's extended spectrum for gram-negative bacteria, specifically *Escherichia coli, Proteus mirabilis, Salmonella* species, and *Haemophilus influenzae*.[6]

The concept of ampicillin's selecting for resistance emanates from a case series published by McDuffie et al.[12] These authors reported 4 cases of *Enterobacteriaceae* chorioamnionitis in gravidas who had received ampicillin prophylaxis for premature rupture of the fetal membranes and GBS carriage. Three of the isolates were *E. coli*. The fourth case was due to *Klebsiella pneumoniae*. Two of the resultant neonates died with fulminant perinatal septicemia. The rationale for publication of their manuscript was the contention that these isolates were examples of "adverse perinatal outcomes due to selection or overgrowth of resistant organisms resulting from the use of ampicillin."

### Induced Resistance

Prolonged use (especially associated with suboptimal dosing) of an antibiotic can select for the emergence of a resistant subpopulation of a previously susceptible strain which then becomes the dominant population. A prime example of this phenomenon was the abuse of kanamycin during the 1970s in newborn intensive care units (NICUs) which resulted in *K. pneumoniae* isolates that were resistant to virtually all the aminoglycosides available at that time. Another prime example of induced resistance is the change in the avidity of binding of penicillin to the penicillin-binding proteins of selected strains of *Streptococcus pneumoniae*.

### Intrinsic Resistance

If an antibiotic is used against a bacterium whose spectrum of susceptibility is not encompassed by that drug, one cannot anticipate having a true biologic effect. Approximately 35–40% of all current *E. coli* isolates are resistant to ampicillin. This resistance is mediated primarily by the presence of significant quantities of β-lactamases within the periplasmic space. More than 95% of all *K. pneumoniae* isolates are similarly inherently resistant to ampicillin. When isolated instances of disease due to *Enterobacteriaceae* occur in the face of ampicillin therapy, the probability is that the same pattern of disease would have been observed had ampicillin not been given. Unless one is dealing with a phenomenon such as anaerobic progression or the use of antibiotics which has an impact on 30S or 50S ribosomes, ineffective antibiotics will not alter the progress of monoetiologic disease.

Drug chemoprophylaxis with ampicillin alters the incidence of disease by diminishing the denominator, thus magnifying the impact of the numerator. As a consequence, perception of the relative importance of an isolate is changed. McDuffie et al.[12] could not adequately approximate the number of cases of perinatal septicemia adequately treated with ampicillin. Therefore, no rigorous risk/benefit analysis can be performed.

### CONCLUSIONS

Because of the limited comparative data available for penicillin G, it is overly assertive to contend that the pharmacokinetic advantages of penicillin G in pregnancy warrant its selection over ampicillin. Similary, the data regarding the spectrum of susceptibility clearly show that ampicillin is the more versatile antibiotic, while the available data on the selection of resistant stains are best regarded as tentative such that validation through prospective study is required. Thus, it appears that ampicillin is superior to penicillin G in the clinical settings of in utero therapy.

### REFERENCES

1. Amstey MS, Gibbs RS: Is penicillin a better choice than ampicillin for prophylaxis of neonatal group B streptococcal infections? Obstet Gynecl 84:1058–1059, 1994.
2. Nau H: Clinical pharmacokinetics in pregnancy and perinatology. II. Penicillins. Dev Pharmacol Ther 10:176–188, 1987.
3. Eleck E, Ivan B, Arr M: Passage of the penicillins from mother to fetus in humans. Int J Clin Pharmacol Ther Toxicol 63:223–228, 1972.
4. Hockert O, Nummi S, Vuopala S, Jarvinen P: Transplacental passage of azidocillin, ampicillin and penicillin to an early and late pregnancy. Scand J Infect Dis 2:125–130, 1970.
5. Campbell B, Cox S: The penicillins. Obstet Gynecol Clin North Am 19:435–447, 1992.
6. Philipson A: Pharmacokinetics of ampicillin during pregnancy. J Infect Dis 136:370–376, 1977.
7. Philipson A: Plasma levels of ampicillin in pregnant women following administration of ampicillin and pivampicillin. Am J Obstet Gynecol 130:674–683, 1978.
8. Mandell GL, Sande MA: Antimicrobial agents: Penicillins, cephalosporins and other beta-lactam antibiotics. In Gilman AG, Rall TW, Nies AS, et al. (eds): The Pharmacological Bases of Therapeutics. 8th ed. Elmsford, NY: Pergamon Press, pp 1080–1085, 1990.
9. MacAulay MA, Abour-Sabe M, Charles D: Transplacental transfer of ampicillin. Am J Obstet Gynecol 96:943–950, 1966.
10. Williams DA: pkA values for some drugs and miscellaneous organic acids and bases. In Foye WO, Lemke TL, Williams DA (eds): Principles of Medicinal Chemistry. 4th ed. Media, PA: Williams & Wilkins, pp 948–961, 1995.
11. Weihrauch TR, Kohler H, Hoffler D: Cerebral toxicity of penicillins in relation to their hydrophobic character. Naunyn-Schmiedebergs Arch Pharmakol 289:55–64, 1975.
12. McDuffie RS, McGregor JA, Gibbs RS: Adverse perinatal outcome and resistant *Enterobacteriaceae* after antibiotic usage for premature rupture of the membranes and group B streptococcus carriage. Obstet Gynecol 82:487–489, 1993.
